# Gender- and Age-Specific Associations of Visit-to-Visit Blood Pressure Variability With Anxiety

**DOI:** 10.3389/fcvm.2021.650852

**Published:** 2021-05-07

**Authors:** Jiandong Zhou, Sharen Lee, Wing Tak Wong, Keith Sai Kit Leung, Ronald Hang Kin Nam, Prudence Shun Hay Leung, Yau-Lam Alex Chau, Tong Liu, Carlin Chang, Bernard Man Yung Cheung, Gary Tse, Qingpeng Zhang

**Affiliations:** ^1^School of Data Science, City University of Hong Kong, Hong Kong, China; ^2^Cardiovascular Analytics Group, Laboratory of Cardiovascular Physiology, Hong Kong, China; ^3^School of Life Sciences, The Chinese University of Hong Kong, Hong Kong, China; ^4^Aston Medical School, Aston University, Birmingham, United Kingdom; ^5^Faculty of Pharmaceutical Sciences, The University of British Columbia, Vancouver, BC, Canada; ^6^Tianjin Key Laboratory of Ionic-Molecular Function of Cardiovascular Disease, Department of Cardiology, Tianjin Institute of Cardiology, Second Hospital of Tianjin Medical University, Tianjin, China; ^7^Division of Neurology, Department of Medicine, The University of Hong Kong, Hong Kong, China; ^8^Division of Clinical Pharmacology and Therapeutics, Department of Medicine, The University of Hong Kong, Hong Kong, China

**Keywords:** blood pressure variability, generalized anxiety disorder, risk prediction, visit-to-visit blood pressure variability, anxiety

## Abstract

**Background:** There is a bidirectional relationship between blood pressure variability (BPV) and anxiety, but few studies have examined the gender- and age-specific effects of visit-to-visit BPV on incident anxiety. We examined the predictive value of BPV for the incidence of anxiety in a family clinic cohort.

**Methods:** Consecutive patients with a first attendance to family medicine clinics in Hong Kong between January 1, 2000, and December 31, 2002, with at least three blood pressure measurements available thereafter were included. The primary endpoint was incident anxiety as identified by ICD-9 coding.

**Results:** This study included 48,023 (50% males) patients with a median follow-up of 224 [interquartile range (IQR): 217–229] months. Females were more likely to develop incident anxiety compared to males (incidence rate: 7 vs. 2%), as were patients of older age. Significant univariate predictors were female gender, older age, preexisting cardiovascular diseases, respiratory diseases, diabetes mellitus, hypertension, and gastrointestinal diseases, various laboratory examinations, and the number of blood pressure measurements. Higher baseline, maximum, minimum, standard deviation (SD), coefficient of variation (CV), and variability score of diastolic blood pressure significantly predicted incident anxiety, as did all systolic blood pressure measures [baseline, latest, maximum, minimum, mean, median, variance, SD, root mean square (RMS), CV, and variability score].

**Conclusions:** The relationships between longer-term visit-to-visit BPV and incident anxiety were identified. Female and older patients with higher blood pressure and higher BPV were at the highest risks of incident anxiety.

## Introduction

Anxiety is a common symptom, and anxiety disorder includes a group of conditions characterized by excessive worry associated with fatigue, restlessness, muscle tension, irritability, sleeping difficulty, and concentration problems. It is a major public health problem in many countries, damaging not only psychological health but also physical health and quality of life. There is a bidirectional relationship between blood pressure variability (BPV) and incident anxiety. The presence of anxiety can exert effects on BPV. Patients with depressive symptoms presented a significantly lower nighttime systolic blood pressure (BP) fall compared with non-depressed patients after controlling for age, sex, and traditional cardiovascular risk factors (1). The control of negative emotions has been shown to influence BP control and BPV (2). Conversely, increased beat-to-beat BPV has been associated with incident anxiety (3). Longer-term visit-to-visit BPV has also been reported as an independent predictor of cognitive impairment in several cohort studies (4–6). With the widespread measurement of BP measurements at home, fluctuations in BP, as well as very high or low BP readings at home, can cause anxiety in patients. However, few previous studies have examined the longitudinal relationship between BPV and anxiety disorders in older cohorts. In this study, we investigated the gender- and age-specific associations of longer-period visit-to-visit BPV with the incidence of anxiety.

## Methods

### Research Design and Data Sources

The study was approved by the Joint Chinese University of Hong Kong–New Territories East Cluster Clinical Research Ethics Committee and Institutional Review Board of the University of Hong Kong/Hospital Authority Hong Kong West Cluster. This was a retrospective cohort study of patients who attended family medicine clinics between January 1, 2000, and March 31, 2002, in the Hong Kong public sector. Patients with at least three BP measurements before being diagnosed with anxiety were included to calculate the variability measures. There were no exclusion criteria. The patients were identified from the Clinical Data Analysis and Reporting System (CDARS), a territory-wide database that centralizes patient information from individual local hospitals to establish comprehensive medical data, including clinical characteristics, disease diagnosis, laboratory results, and medication prescription details. The system has been previously used by both our team and other teams in Hong Kong to conduct studies on comparative drug action (7), specific diseases (8–10), model development (11), or visit-to-visit variability in metabolic parameters (12, 13). Data were obtained regarding consecutive patients diagnosed with anxiety, excluding those who died or discharged within 24 h after the first diastolic/systolic BP measurement and those with fewer than three diastolic/systolic BP measurements (study baseline). Mortality data were obtained from the Hong Kong Death Registry, a population-based official government registry with the registered death records of all Hong Kong citizens. Data on the clinical characteristics, disease diagnosis, laboratory results (including complete blood count, renal and liver function tests, glycemic and lipid profiles, and diastolic/systolic BP), and medication prescription details were extracted. Patients with anxiety were identified with the diagnosis codes 311, 296.3, 296.2, 308, 300.4, 292.84, 298, 300.02, 291.89, 293.84, 292.89, 294.9, 300.2, 309.24, 300.01, 309.21. The ICD-9 codes for past comorbidities and historical medication prescriptions are detailed in the Supplementary Material.

### Primary Outcome and Statistical Analysis

The primary outcome was incident cases of anxiety from the study baseline in a time-to-event analysis. Follow-up was carried out until December 31, 2019. We extracted the baseline/latest/maximum/minimum values of diastolic and systolic BPs and calculated the temporal variability measures of diastolic and systolic BPs: (1) mean, (2) median, (3) standard deviation (SD), (4) root mean square (RMS) by first squaring all BP values and then calculating the square root of the mean of the squares, (5) coefficient of variation (CV) by dividing the BP SD by the mean BP and then multiplying by 100, and (6) a variability score [from 0 (low) to 100 (high)] defined as the number of changes in BP of 5 mmHg or more, i.e., 100 × (number of absolute BP change of each two successive measurements > 5)/number of measurements.

Clinical characteristics were summarized using statistical descriptive statistics. Continuous variables were presented as median [95% confidence interval (CI) or interquartile range (IQR)], and categorical variables were presented count (%). The Mann–Whitney *U*-test was used to compare continuous variables. The χ^2^-test with Yates' correction was used for 2 × 2 contingency data, and Pearson's χ^2^-test was used for contingency data for variables with more than two categories. Univariate Cox regression models were conducted based on cohorts of males and females, respectively, to identify the significant predictors of anxiety. Significant univariate predictors of demographics, prior comorbidities, clinical and biochemical tests, medication prescriptions, and BPV were used as input of a multivariate Cox analysis model, adjusted for demographics and comorbidities. Hazard ratios (HRs) with corresponding 95% CIs and *p*-values were reported. All significance tests were two-tailed and considered significant if *p*-values < 0.001. Data analysis was performed using the RStudio software (version: 1.1.456) and Python (version: 3.6).

## Results

### Baseline Clinical Characteristics and Anxiety Incidence

This study included a total of 48,023 (50% males) patients with a median follow-up of 224 (IQR: 217–229) months (Supplementary Figure 1). Among the 23,964 male patients, 495 (incidence rate: 2.1%, median age: 70 [IQR: 57–79] years old) developed anxiety. By contrast, females had a higher incidence rate, with 1,687 of 24,059 (incidence rate: 7.0%, median age: 68 [IQR: 56–78] years old) developing anxiety.

The clinical characteristics of the included patients are provided in Table 1. Compared with female patients, male patients were more likely to suffer from cardiovascular diseases (63.63 vs. 52.97%, *p* < 0.0001), kidney disease (29.89 vs. 17.10%, *p* < 0.0001), and stroke (38.38 vs. 28.30%, *p* < 0.0001). They were more likely to be prescribed angiotensinogen-converting enzyme inhibitors (ACEI) (18.18 vs. 11.20%) and other antihypertensive drugs (23.03 vs. 4.82%) than female patients.

**Table 1:** Clinical characteristics of patients included in this cohort.

**Characteristics**	**Males (*N* = 23,964; event: 495, incidence rate: 2.07%; mortality: 195, 39.4%)**	**Females (*N* = 24,059; event: 1,687, incidence rate: 7.01%; mortality: 431, 25.68%)**	***p***
	**Median (IQR); Max; N or Count (%)**	**Median (IQR); Max; N or Count (%)**	
**Demographics**			
Age of first BP test, years	61.4 (50.8–69.8); *n* = 495	59.3 (49.3–69.3); *n* = 1,678	0.1453
**Past comorbidity**			
Cardiovascular	347 (70.10%)	1,209 (72.05%)	0.2253
Respiratory	315 (63.63%)	889 (52.97%)	<0.0001^***^
Kidney	148 (29.89%)	287 (17.10%)	<0.0001^***^
Endocrine	25 (5.05%)	83 (4.94%)	0.2588
Diabetes mellitus	79 (15.95%)	287 (17.10%)	0.1086
Hypertension	336 (67.87%)	1,131 (67.40%)	0.3494
Gastrointestinal	275 (55.55%)	942 (56.13%)	0.2378
Obesity	3 (0.60%)	6 (0.35%)	0.8561
Stroke	190 (38.38%)	475 (28.30%)	<0.0001^***^
**Medications**			
ACEI	90 (18.18%)	188 (11.20%)	0.0025^**^
ARB	2 (0.40%)	9 (0.53%)	0.8262
Calcium channel blockers	140 (28.28%)	379 (22.58%)	0.1477
Beta blockers	156 (31.51%)	537 (32.00%)	0.5235
Diuretics for heart failure	14 (2.82%)	51 (3.03%)	0.5597
Diuretics for hypertension	59 (11.91%)	197 (11.74%)	0.2936
Nitrates	74 (14.94%)	203 (12.09%)	0.8393
Antihypertensive drugs	114 (23.03%)	81 (4.82%)	<0.0001^***^
Antidiabetic drugs	54 (10.90%)	175 (10.42%)	0.5855
Statins and fibrates	75 (15.15%)	258 (15.37%)	0.6512
**Complete blood count**			
Mean corpuscular volume, fl	91.3 (88.3–94.0); *n* = 242	89.4 (86.2–92.5); *n* = 889	0.1994
Basophil, ×10^9^/L	0.04 (0.02–0.046); *n* = 113	0.03 (0.01–0.03); *n* = 390	0.35
Eosinophil, ×10^9^/L	0.1 (0.1–0.2); *n* = 132	0.1 (0.1–0.2); *n* = 485	0.3711
Lymphocyte, ×10^9^/L	1.78 (1.3–2.2); *n* = 133	1.8 (1.4–2.4); *n* = 489	0.1135
Monocyte, ×10^9^/L	0.5 (0.4–0.7); *n* = 132	0.4 (0.3–0.6); *n* = 486	0.6279
Neutrophil, ×10^9^/L	4.3 (3.5–6.55); *n* = 132	4.1 (3.2–5.53); *n* = 485	0.6455
White cell count, ×10^9^/L	7.16 (6.0–8.95); *n* = 243	6.8 (5.6–8.3); *n* = 892	0.787
Mean corpuscular hemoglobin, pg	31.1 (29.85–32.1); *n* = 242	30.5 (29.1–31.5); *n* = 889	0.8937
Platelet, ×10^9^/L	229.0 (191.0–265.0); *n* = 243	244.0 (209.0–293.0); *n* = 891	0.431
Reticulocyte, ×10^9^/L	31.9 (28.8–60.9); *n* = 3	60.9 (44.0–91.77); *n* = 17	<0.0001^***^
Red cell count, ×10^12^/L	4.65 (4.34–5.01); *n* = 241	4.31 (4.04–4.6); *n* = 889	0.3762
Hematocrit, L/L	0.43 (0.4–0.45); *n* = 226	0.38 (0.4–0.4); *n* = 836	0.9949
**Renal and liver function tests**			
Potassium, mmol/L	4.15 (3.89–4.5); *n* = 337	4.2 (3.9–4.47); *n* = 1,041	0.297
Urate, mmol/L	0.398 (0.35–0.475); *n* = 86	0.32 (0.26–0.39); *n* = 253	0.1253
Albumin, g/L	42.25 (40.0–44.368); *n* = 290	42.0 (39.8–44.0); *n* = 941	0.9327
Sodium, mmol/L	141.0 (139.0–142.0); *n* = 337	141.0 (139.0–142.0); *n* = 1,041	0.5236
Urea, mmol/L	5.7 (4.835–6.8); *n* = 336	5.3 (4.3–6.3); *n* = 1,039	0.9642
Protein, g/L	74.0 (70.69–77.35); *n* = 287	74.0 (71.0–78.0); *n* = 939	0.6126
Creatinine, μmol/L	98.0 (88.0–111.0); *n* = 337	75.0 (67.0–85.0); *n* = 1,041	<0.0001^***^
Alkaline phosphatase, U/L	75.0 (60.84–92.0); *n* = 248	77.0 (60.0–93.5); *n* = 815	0.7759
Aspartate transaminase, U/L	23.0 (17.0–27.0); *n* = 68	21.0 (18.0–26.0); *n* = 214	0.544
Alanine transaminase, U/L	23.0 (17.0–35.0); *n* = 225	19.0 (14.0–27.0); *n* = 740	0.0023^**^
Bilirubin, μmol/L	10.0 (7.5–13.0); *n* = 250	9.0 (6.3874–11.3); *n* = 824	0.3542
**Glycemic and lipid profiles**			
Triglyceride, mmol/mol	1.6 (1.1–2.3); *n* = 156	1.4 (0.97–2.0); *n* = 546	0.6491
LDL, mmol/mol	3.3 (2.8–3.7); *n* = 116	3.2 (2.6–3.9); *n* = 373	0.3887
HDL, mmol/mol	1.2 (1.02–1.4); *n* = 118	1.4 (1.195–1.66); *n* = 390	0.3572
HbA1c, g/dl	13.9 (12.9–15.1); *n* = 215	12.8 (11.7–13.6); *n* = 788	0.0031^**^
Cholesterol, mmol/L	5.2 (4.6–5.7); *n* = 156	5.4 (4.8–6.1); *n* = 548	0.128
Fasting glucose, mmol/L	5.8 (5.2–7.5); *n* = 240	5.6 (5.1–7.0); *n* = 787	0.9374
**Diastolic blood pressure measurements**			
Number of measurements	7 (5–10); *n* = 495	7 (6–10); *n* = 1,678	0.1225
Baseline, mmHg	77.0 (68.0–84.5); *n* = 495	72.0 (65.0–80.0); *n* = 1,678	0.041^*^
Latest, mmHg	74.0 (66.0–81.0); *n* = 495	70.0 (63.0–78.0); *n* = 1,678	0.6643
Maximum, mmHg	89.0 (81.5–97.0); *n* = 495	86.0 (79.0–93.0); *n* = 1,678	0.8432
Minimal, mmHg	63.0 (56.0–70.0); *n* = 495	58.0 (52.0–66.0); *n* = 1,678	0.0323^*^
Mean, mmHg	75.4 (69.8–81.6); *n* = 495	71.9 (66.2–77.2); *n* = 1,678	0.0065^**^
Median, mmHg	75.5(69.5–81.0); *n* = 495	71.5 (66.0–77.0); *n* = 1,678	0.0234^*^
Variance	54.3 (31.4–88.9); *n* = 495	56.1 (33.4–84.3); *n* = 1,678	0.2326
SD	7.4 (5.6–9.4); *n* = 495	7.5 (5.8–9.2); *n* = 1,678	0.5344
RMS	75.9 (70.3–81.9); *n* = 495	72.3 (66.6–77.6); *n* = 1,678	0.0422^*^
CV	0.09 (0.07–0.12); *n* = 495	0.1001 (0.073–0.1); *n* = 1,678	<0.0001^***^
Variability score	57.1 (46.2–66.7); *n* = 495	55.8 (47.4–66.2); *n* = 1,678	0.5416
**Systolic blood pressure measurements**			
Number of measurements			
Baseline, mmHg	135.0 (120.0–150.0); *n* = 495	132.0 (117.0–147.0); *n* = 1,678	0.1594
Latest, mmHg	132.0 (121.0–143.0); *n* = 495	131.0 (119.0–142.0); *n* = 1,678	0.7247
Maximum, mmHg	156.0 (144.0–169.0); *n* = 495	157.0 (140.0–173.0); *n* = 1,678	0.7289
Minimal, mmHg	111.0 (102.0–121.0); *n* = 495	109.0 (101.0–120.0); *n* = 1,678	0.1322
Mean, mmHg	134.0 (125.4–142.4); *n* = 495	132.5 (123.3–141.0); *n* = 1,678	0.3252
Median, mmHg	133.0 (125.0–142.0); *n* = 495	132.0 (122.5–141.0); *n* = 1,678	0.2253
Variance	165.7 (96.9–255.0); *n* = 495	161.8 (91.7–256.7); *n* = 1,678	0.1806
SD	12.9 (9.8–15.9); *n* = 495	12.7 (9.6–16.0); *n* = 1,678	0.2178
RMS	134.7 (126.2–143.0); *n* = 495	133.4 (124.0–141.9); *n* = 1,678	0.2509
CV	0.09 (0.07–0.11); *n* = 495	0.09 (0.07–0.12); *n* = 1,678	0.2264
Variability score	70.0 (60.0–77.0); *n* = 495	70.6 (57.1–78.6); *n* = 1,678	0.7872

*ACEI, angiotensinogen-converting enzyme inhibitor; ARB, angiotensin receptor blocker; LDL, low-density lipoprotein cholesterol; HDL, high-density lipoprotein cholesterol; SD, standard deviation; RMS, root mean square; CV, coefficient of variation*.

* *p ≤ 0.05*,

** *p ≤ 0.01*,

*** *p ≤ 0.001*.

Nevertheless, female patients had a higher reticulocyte level (median: 60.9, IQR: 44.0–91.77 vs. median: 31.9, IQR: 28.8–60.9, *p* < 0.0001), lower creatinine level (median: 75.0, IQR: 67.0–85.0 vs. median: 98.0, IQR: 88.0–111.0, *p* < 0.0001), lower alanine transaminase amount (median: 19.0, IQR: 14.0–27.0 vs. median: 23.0, IQR: 17.0–35.0, *p* = 0.0023), and lower HbA1c level (median: 12.8, IQR: 11.7–13.6 vs. median: 13.9, IQR: 12.9–15.1, *p* = 0.0031). Regarding diastolic BP measurements, female patients had a lower mean (median: 58.0, IQR: 52.0–66.0 vs. median: 63.0, IQR: 56.0–70.0, *p* = 0.0323), median (median: 71.9, IQR: 66.2–77.2 vs. median: 75.4, IQR: 69.8–81.6, *p* = 0.0065), RMS (median: 72.3, IQR: 66.6–77.6 vs. median: 75.9, IQR: 70.3–81.9, *p* = 0.0422), and CV (median: 0.1001, IQR: 0.073–0.1 vs. median: 0.09, IQR: 0.07–0.12, *p* < 0.0001).

### Incidence of Anxiety on Follow-Up and Significant Predictors

**Figure 1:**
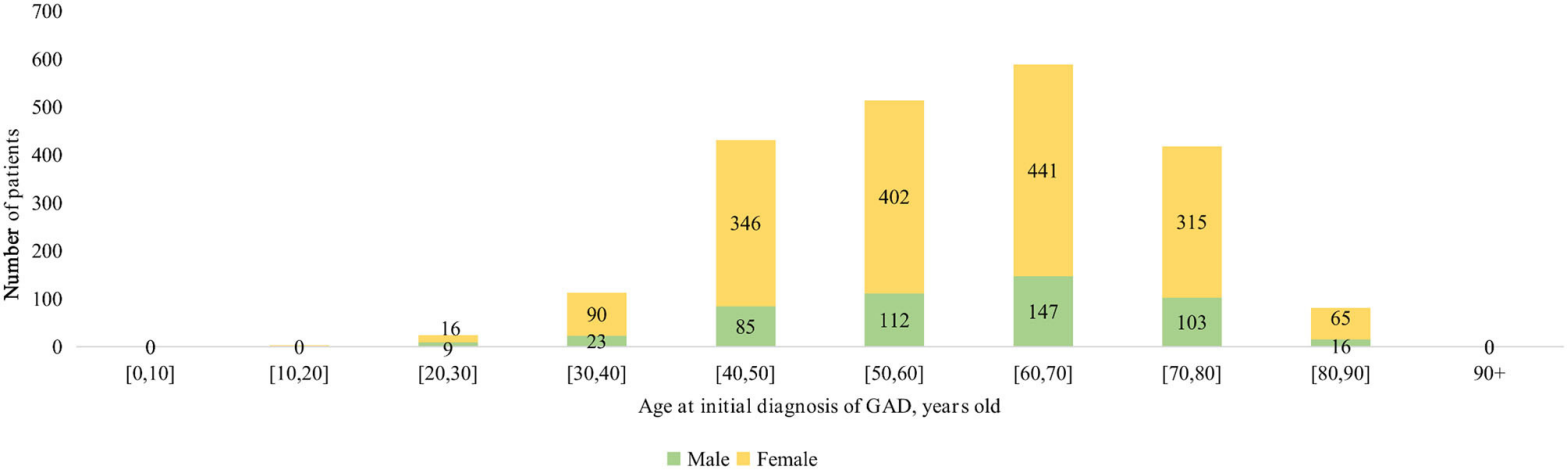
Age-specific incidence of anxiety among male and female subgroups.

**Figure 2:**
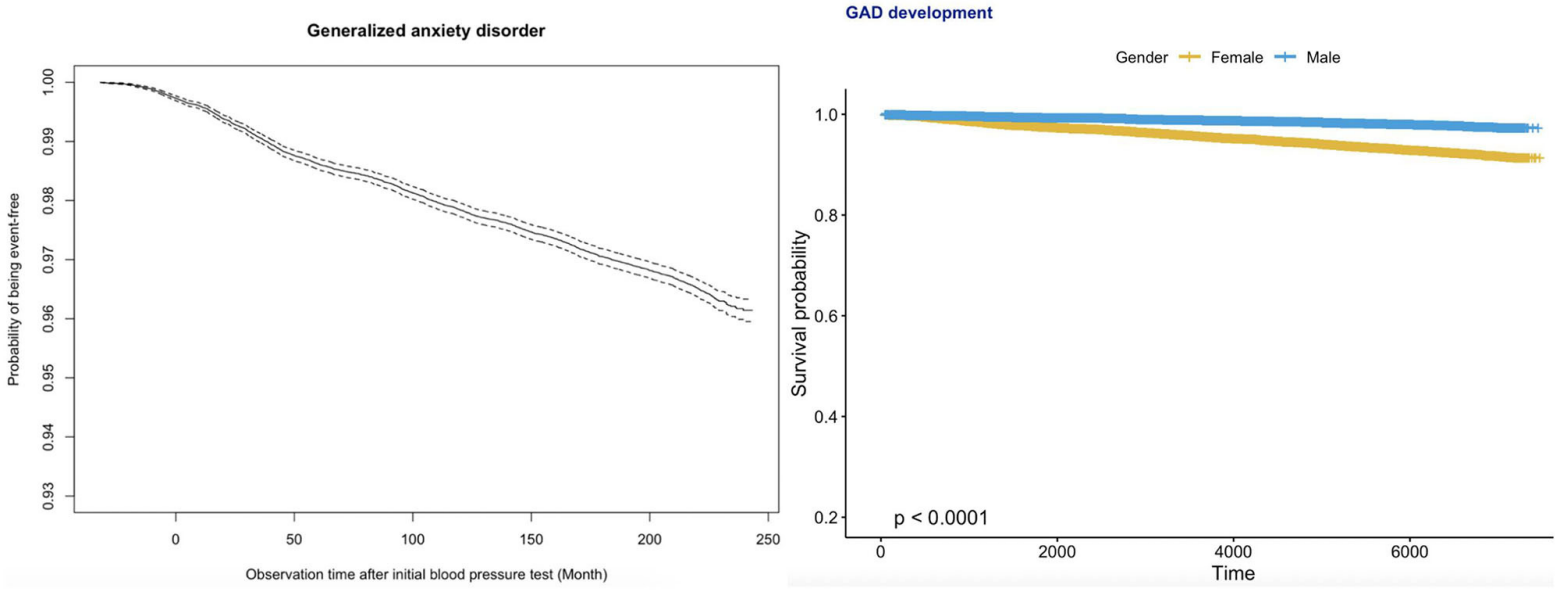
Kaplan–Meier survival curves of incident anxiety development for the whole cohort (top) and stratified by gender (bottom).

The age-specific incidences of anxiety among male and female subgroups are shown in Figure 1. The number of female patients developing anxiety was more than double that of male patients among those over 30 years of age. Kaplan–Meier survival curves in Figure 2 show that females had a higher risk of developing anxiety than males.

Univariate Cox regression demonstrated the following significant predictors for incident anxiety: demographics, namely, gender [female as comparison: HR for male: 0.30, 95% CI: [0.27, 0.33], *p* < 0.0001^***^] and older age [HR: 1.23, 95% CI: [1.19, 2.03], *p* < 0.0001]. The specific risks differed between age groups: [40, 50] years old [HR: 1.42, 95% CI: [1.27, 1.57], *p* < 0.0001], [50, 60] years old [HR: 1.30, 95% CI: [1.18, 1.43], *p* < 0.0001], [60, 70] years old [HR: 1.11, 95% CI: [1.01, 1.22], *p* = 0.0008], [70, 80] years old [HR: 1.71, 95% CI: [1.64, 1.79], *p* < 0.0001], [80, 90] years old [HR: 1.46, 95% CI: [1.37, 1.57], *p* < 0.0001]; past history of cardiovascular diseases [HR: 4.00, 95% CI: [3.64, 4.39], *p* < 0.0001], respiratory diseases [HR: 1.33, 95% CI: [1.22, 1.45], *p* < 0.0001], diabetes mellitus [HR: 1.17, 95% CI: [1.05, 1.31], *p* = 0.0062], hypertension [HR: 1.17, 95% CI: [1.07, 1.28], *p* = 0.0008], and gastrointestinal disorders [HR: 1.90, 95% CI: [1.74, 2.07], *p* < 0.0001]; laboratory parameters, namely, lower neutrophil [HR: 0.35, 95% CI: [0.24, 0.50], *p* < 0.0001], less white cell count [HR: 0.92, 95% CI: [0.89, 0.95], *p* < 0.0001], lower mean corpuscular hemoglobin level [HR: 0.94, 95% CI: [0.92, 0.96], *p* < 0.0001], higher red cell count [HR: 1.001, 95% CI: [1.001, 1.002], *p* = 0.0002], lower urate level [HR: 0.03, 95% CI: [0.01, 0.07], *p* < 0.0001], higher albumin level [HR: 1.04, 95% CI: [1.03, 1.06], *p* < 0.0001], lower urea level [HR: 0.87, 95% CI: [0.85, 0.90], *p* < 0.0001], lower creatinine level [HR: 0.98, 95% CI: [0.979, 0.982], *p* < 0.0001], lower alkaline phosphatase level [HR: 0.995, 95% CI: [0.993, 0.997], *p* < 0.0001], lower bilirubin level [HR: 0.97, 95% CI: [0.95, 0.98], *p* < 0.0001], higher high-density lipoprotein (HDL) level [HR: 1.54, 95% CI: [1.25, 1.91], *p* = 0.0001], and lower fasting glucose level [HR: 0.96, 95% CI: [0.93, 0.98], *p* = 0.0007]; diastolic BP measures, namely, higher baseline value [HR: 1.49, 95% CI: [1.08, 2.45], *p* < 0.0001], higher maximum value [HR: 1.19, 95% CI: [1.06, 1.54], *p* < 0.0001], higher minimum value [HR: 1.23, 95% CI: [1.08, 2.06], *p* < 0.0001], larger SD [HR: 1.18, 95% CI: [1.03, 1.95], *p* = 0.0008], larger CV [HR: 1.13, 95% CI: [1.05, 1.38], *p* = 0.0002], and larger variability score [HR: 1.16, 95% CI: [1.04, 1.85], *p* = 0.0003]; and larger values of all systolic BP measures (baseline, latest, maximum, minimum, mean, median, variance, SD, RMS, CV, and variability score) (HR: >1, *p* < 0.001).

**Figure 3:**
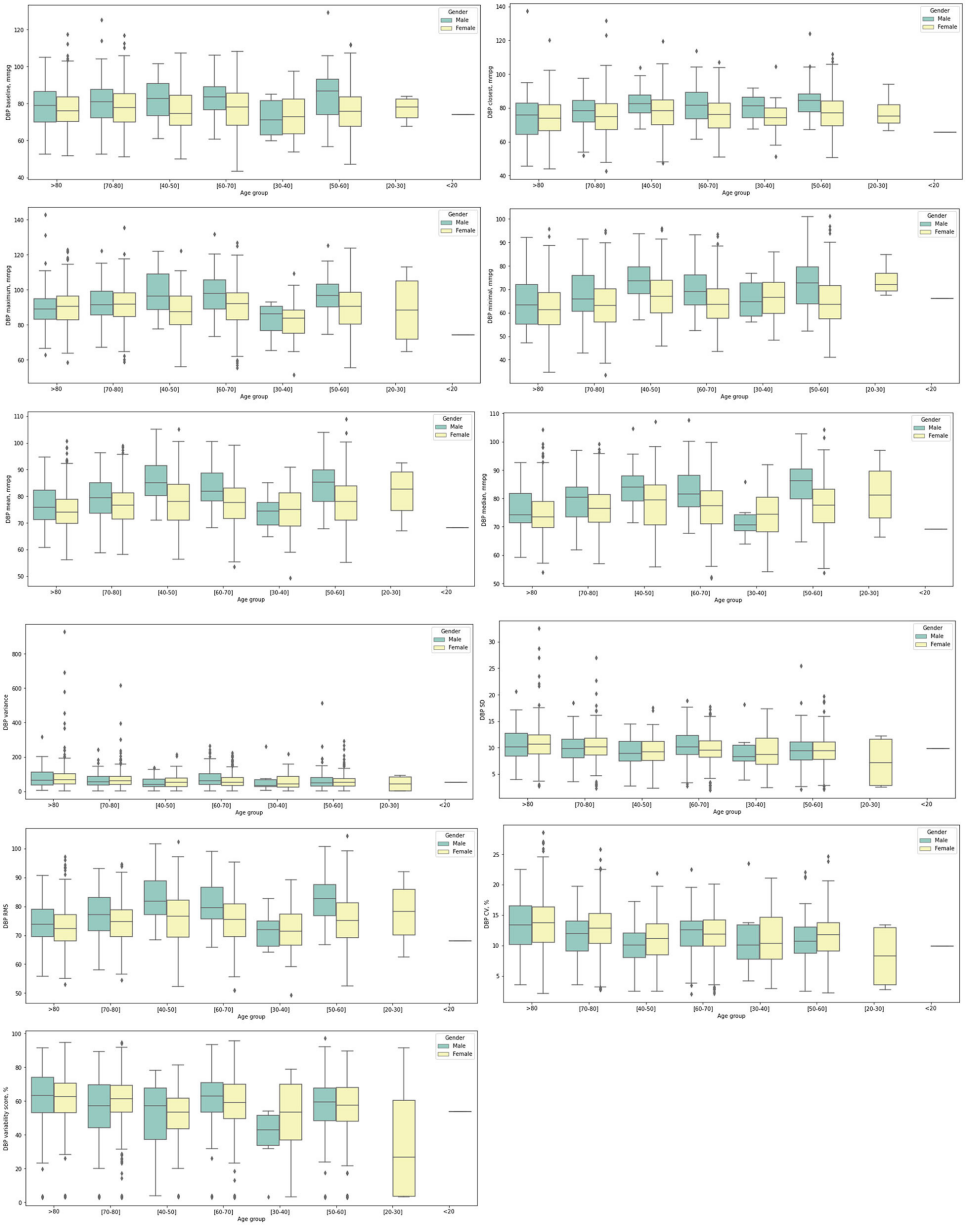
Box plots showing diastolic blood pressure (DBP) measurements stratified by gender and age at anxiety presentation.

**Figure 4:**
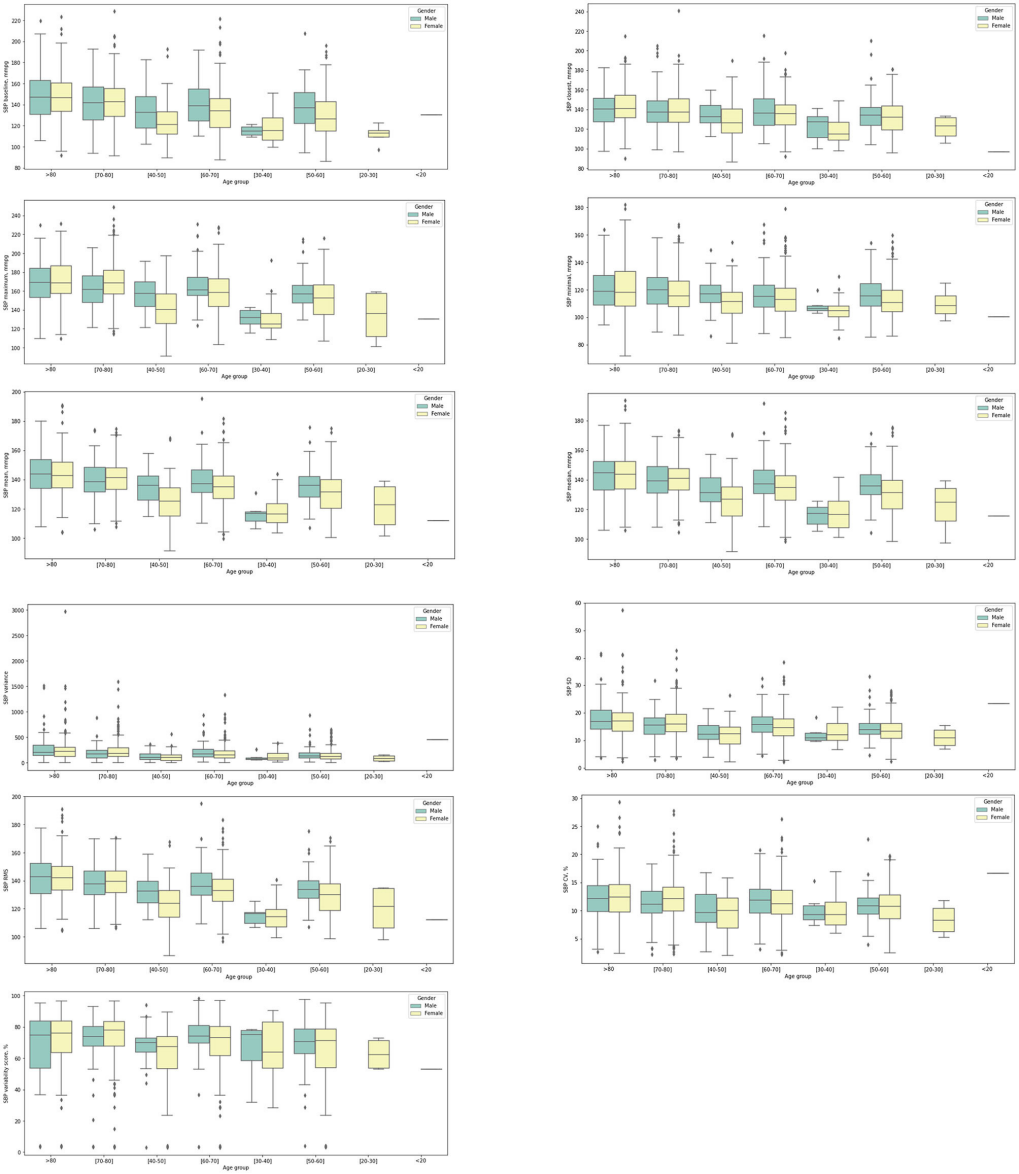
Box plots showing systolic blood pressure (SBP) measurements stratified by gender and age at anxiety presentation.

In addition, the significant univariate predictors of all-cause mortality after anxiety presentation were also identified (Table 2). Significant univariate predictors (*p* < 0.05) were entered into a multivariate Cox regression model, with most of the above univariate predictors remaining significant (Table 3). Next, we further analyzed different BP values in patients who developed anxiety with age stratification (Figures 3, 4 and Supplementary Table 2). There is an age-related increase in mean, median, and measures of variability for both diastolic and systolic BPs.

**Table 2:** Univariate Cox analysis to predict incident anxiety and mortality.

**Characteristics**	**Anxiety (*N* = 2,173)**	***P***	**Mortality (*N* = 626)**	***p***
	**HR [95% CI]**		**HR [95% CI]**	
**Demographics**				
Male gender	0.30 [0.27, 0.33]	<0.0001^***^	1.74 [1.69, 1.79]	<0.0001^***^
Age at first blood pressure test, years	1.23 [1.19, 2.03]	<0.0001^***^	1.10 [1.10, 1.11]	<0.0001^***^
[0, 10]	-	-	-	-
[10, 20]	0.31 [0.10, 0.97]	0.0446^*^	0.01 [0.00, 0.07]	<0.0001^***^
[20, 30]	0.54 [0.36, 0.79]	0.0019^**^	0.13 [0.10, 0.17]	<0.0001^***^
[30, 40]	1.10 [0.91, 1.33]	0.3140	0.06 [0.05, 0.08]	<0.0001^***^
[40, 50]	1.42 [1.27, 1.57]	<0.0001^***^	0.11 [0.10, 0.12]	<0.0001^***^
[50, 60]	1.30 [1.18, 1.43]	<0.0001^***^	0.25 [0.24, 0.27]	<0.0001^***^
[60, 70]	1.11 [1.01, 1.22]	0.0008^**^	0.97 [0.94, 1.00]	0.0749
[70, 80]	1.71 [1.64, 1.79]	<0.0001^***^	3.51 [3.41, 3.61]	<0.0001^***^
[80, 90]	1.46 [1.37, 1.57]	<0.0001^***^	6.00 [5.78, 6.23]	<0.0001^***^
90+	-	0.9640	8.76 [7.75, 9.89]	<0.0001^***^
**Past comorbidity**				
Cardiovascular	4.00 [3.64, 4.39]	<0.0001^***^	2.18 [2.12, 2.24]	<0.0001^***^
Respiratory	1.33 [1.22, 1.45]	<0.0001^***^	3.90 [3.77, 4.02]	<0.0001^***^
Kidney	0.87 [0.78, 0.96]	0.0076^**^	2.15 [2.09, 2.22]	<0.0001^***^
Endocrine	1.12 [0.92, 1.36]	0.2500	2.08 [1.97, 2.20]	<0.0001^***^
Diabetes mellitus	1.17 [1.05, 1.31]	0.0062^**^	0.96 [0.92, 1.00]	0.0385^*^
Hypertension	1.17 [1.07, 1.28]	0.0008^***^	0.87 [0.84, 0.90]	<0.0001^***^
Gastrointestinal	1.90 [1.74, 2.07]	<0.0001^***^	1.20 [1.17, 1.23]	<0.0001^***^
Obesity	2.07 [1.08, 3.98]	0.0296^*^	0.39 [0.25, 0.62]	<0.0001^***^
Stroke	1.07 [0.98, 1.17]	0.1410	1.94 [1.89, 2.00]	<0.0001^***^
**Medications**				
ACEI	0.66 [0.59, 0.75]	<0.0001^***^	1.80 [1.74, 1.86]	<0.0001^***^
ARB	0.80 [0.44, 1.45]	0.4600	1.50 [1.29, 1.75]	<0.0001^***^
Calcium channel blockers	0.70 [0.64, 0.78]	<0.0001^***^	1.85 [1.80, 1.91]	<0.0001^***^
Beta blockers	1.20 [1.10, 1.32]	0.0001^***^	1.06 [1.03, 1.10]	0.0001^***^
Diuretics for heart failure	0.67 [0.52, 0.85]	0.0013^**^	3.98 [3.79, 4.18]	<0.0001^***^
Diuretics for hypertension	0.84 [0.73, 0.95]	0.0073^**^	1.40 [1.35, 1.45]	<0.0001^***^
Nitrates	1.05 [0.93, 1.20]	0.4130	1.91 [1.84, 1.98]	<0.0001^***^
Antihypertensive drugs	0.72 [0.62, 0.84]	<0.0001^***^	2.23 [2.15, 2.31]	<0.0001^***^
Antidiabetic drugs	0.59 [0.51, 0.67]	<0.0001^***^	1.40 [1.35, 1.45]	<0.0001^***^
Statins and fibrates	1.11 [0.98, 1.24]	0.0908	1.06 [1.02, 1.10]	0.0068^**^
**Complete blood count**				
Mean corpuscular volume, fl	0.995 [0.988, 1.002]	0.1930	1.021 [1.018, 1.024]	<0.0001^***^
Basophil, ×10^9^/L	0.20 [0.02, 1.68]	0.1370	2.00 [1.05, 3.79]	0.0339^*^
Eosinophil, ×10^9^/L	0.81 [0.54, 1.22]	0.3160	1.11 [1.02, 1.21]	0.0178^*^
Lymphocyte, ×10^9^/L	1.03 [1.01, 1.06]	0.0161^*^	0.66 [0.63, 0.68]	<0.0001^***^
Monocyte, ×10^9^/L	1.88 [0.33, 10.83]	0.4800	1.04 [0.65, 1.66]	0.871
Neutrophil, ×10^9^/L	0.35 [0.24, 0.50]	<0.0001^***^	2.10 [1.96, 2.26]	<0.0001^***^
White cell count, ×10^9^/L	0.92 [0.89, 0.95]	<0.0001^***^	1.08 [1.08, 1.09]	<0.0001^***^
Mean corpuscular hemoglobin, pg	0.94 [0.92, 0.96]	<0.0001^***^	1.06 [1.06, 1.06]	<0.0001^***^
Platelet, ×10^9^/L	1.00 [0.98, 1.01]	0.6170	1.03 [1.03, 1.04]	<0.0001^***^
Reticulocyte, ×10^9^/L	11.70 [0.38, 357.50]	0.1590	1.78 [0.89, 3.56]	0.105
Red cell count, ×10^12^/L	1.001 [1.001, 1.002]	0.0002^***^	0.998 [0.998, 0.998]	<0.0001^***^
Hematocrit, L/L	1.003 [0.993, 1.013]	0.5270	0.999 [0.996, 1.001]	0.312
Basophil, ×10^9^/L	0.97 [0.88, 1.06]	0.4620	0.57 [0.55, 0.59]	<0.0001^***^
Eosinophil, ×10^9^/L	0.63 [0.19, 2.07]	0.4490	0.50 [0.12, 0.81]	<0.0001^***^
**Renal and liver function tests**				
Potassium, mmol/L	0.88 [0.79, 0.97]	0.0102^*^	0.95 [0.92, 0.98]	0.0026 ^**^
Urate, mmol/L	0.03 [0.01, 0.07]	<0.0001^***^	10.65 [8.23, 13.77]	<0.0001^***^
Albumin, g/L	1.04 [1.03, 1.06]	<0.0001^***^	0.88 [0.87, 0.88]	<0.0001^***^
Sodium, mmol/L	1.02 [1.00, 1.04]	0.0196^*^	0.948 [0.943, 0.952]	<0.0001^***^
Urea, mmol/L	0.87 [0.85, 0.90]	<0.0001^***^	1.111 [1.108, 1.114]	<0.0001^***^
Protein, g/L	1.01 [1.00, 1.02]	0.0528	0.95 [0.95, 0.95]	<0.0001^***^
Creatinine, μmol/L	0.98 [0.979, 0.982]	<0.0001^***^	1.003 [1.003, 1.003]	<0.0001^***^
Alkaline phosphatase, U/L	0.995 [0.993, 0.997]	<0.0001^***^	1.002 [1.002, 1.002]	<0.0001^***^
Aspartate transaminase, U/L	1.000 [0.998, 1.001]	0.6580	1.001 [1.000, 1.001]	<0.0001^***^
Alanine transaminase, U/L	0.999 [0.997, 1.001]	0.4250	0.998 [0.997, 0.999]	<0.0001^***^
Bilirubin, μmol/L	0.97 [0.95, 0.98]	<0.0001^***^	1.007 [1.005, 1.009]	<0.0001^***^
**Glycemic and lipid profiles**				
Triglyceride, mmol/mol	0.98 [0.92, 1.04]	0.4300	0.97 [0.95, 0.99]	0.0024 ^**^
LDL, mmol/mol	1.03 [0.93, 1.13]	0.6050	0.91 [0.88, 0.95]	<0.0001^***^
HDL, mmol/mol	1.54 [1.25, 1.91]	0.0001^***^	0.82 [0.75, 0.90]	<0.0001^***^
HbA1c, g/dl	1.01 [1.00, 1.03]	0.0613	0.98 [0.98, 0.98]	<0.0001^***^
Cholesterol, mmol/L	1.04 [0.98, 1.11]	0.2230	0.92 [0.90, 0.94]	<0.0001^***^
Fasting glucose, mmol/L	0.96 [0.93, 0.98]	0.0007^***^	1.05 [1.05, 1.06]	<0.0001^***^
**Diastolic blood pressure measurements**				
Number of measurements	0.89 [0.18, 1.23]	0.1352	0.53 [0.42, 1.71]	0.2342
Baseline, mmHg	1.49 [1.08, 2.45]	<0.0001^***^	1.03 [1.01, 1.24]	<0.0001^***^
Latest, mmHg	1.001 [0.997, 1.004]	0.6870	1.07 [1.02, 1.28]	<0.0001^***^
Maximum, mmHg	1.19 [1.06, 1.54]	<0.0001^***^	1.04 [1.02, 1.08]	<0.0001^***^
Minimal, mmHg	1.23 [1.08, 2.06]	<0.0001^***^	1.09 [1.01, 1.2]	0.0442^*^
Mean, mmHg	1.03 [1.01, 1.39]	0.0011^**^	1.1 [1.03, 1.7]	<0.0001^***^
Median, mmHg	1.04 [1.01, 1.53]	0.0026^**^	1.28 [1.16, 1.98]	<0.0001^***^
Variance	1.002 [1.001, 1.003]	0.0491^*^	1.003 [1.003, 1.003]	<0.0001^***^
SD	1.18 [1.03, 1.95]	0.0008^***^	1.06 [1.05, 1.06]	<0.0001^***^
RMS	1.23 [1.09, 1.6]	0.0078^**^	1.18 [1.02, 1.93]	<0.0001^***^
CV	1.13 [1.05, 1.38]	0.0002^***^	40.89 [28.15, 59.38]	<0.0001^***^
Variability score	1.16 [1.04, 1.85]	0.0003^***^	1.001 [1.000, 1.002]	0.0082^**^
**Systolic blood pressure measurements**				
Number of measurements				
Baseline, mmHg	1.25 [1.04, 1.93]	<0.0001^***^	1.015 [1.014, 1.015]	<0.0001^***^
Latest, mmHg	1.34 [1.12, 1.88]	<0.0001^***^	1.009 [1.008, 1.010]	<0.0001^***^
Maximum, mmHg	1.15 [1.04, 1.33]	<0.0001^***^	1.009 [1.009, 1.010]	<0.0001^***^
Minimal, mmHg	1.04 [1.01, 1.07]	<0.0001^***^	1.022 [1.021, 1.023]	<0.0001^***^
Mean, mmHg	1.26 [1.03, 1.56]	<0.0001^***^	1.031 [1.030, 1.032]	<0.0001^***^
Median, mmHg	1.16 [1.04, 1.29]	<0.0001^***^	1.030 [1.029, 1.031]	<0.0001^***^
Variance	1.04 [1.01, 1.1]	<0.0001^***^	1.001 [1.001, 1.001]	<0.0001^***^
SD	1.17 [1.07, 1.85]	<0.0001^***^	1.061 [1.059, 1.063]	<0.0001^***^
RMS	1.59 [1.18, 1.99]	<0.0001^***^	1.031 [1.030, 1.032]	<0.0001^***^
CV	1.07 [1.02, 1.23]	<0.0001^***^	629.90 [424.60, 934.40]	<0.0001^***^
Variability score	1.26 [1.04, 1.82]	0.0008 ^***^	1.000 [0.999, 1.001]	0.8170

*ACEI, angiotensinogen-converting enzyme inhibitor; ARB, angiotensin receptor blocker; LDL, low-density lipoprotein cholesterol; HDL, high-density lipoprotein cholesterol; SD, standard deviation; RMS, root mean square; CV, coefficient of variation*.

* *p ≤ 0.05*,

** *p ≤ 0.01*,

*** *p ≤ 0.001*.

**Table 3:** Multivariate Cox regression analysis to predict incident anxiety.

	**HR [95% CI]**	***Z*-value**	***p***
**Demographics**			
Male gender	0.23 [0.11, 0.48]	−3.93	0.0001^***^
Age at first blood pressure test, years	1.04 [1.02, 1.07]	0.15	0.0029^**^
[20, 30]	1.24 [1.26, 5.98]	0.27	0.0019^**^
[30, 40]	1.07 [1.02, 2.14]	0.56	0.0034^**^
[40, 50]	1.98 [1.38, 10.24]	0.82	<0.0001^***^
[50, 60]	1.47 [1.22, 9.92]	0.39	0.0056^**^
[60, 70]	1.48 [1.05, 4.88]	0.63	<0.0001^***^
[70, 80]	1.06 [1.01, 16.38]	0.004	0.0069^**^
**Past comorbidities**			
Cardiovascular	3.69 [1.99, 6.84]	4.16	<0.0001^***^
Respiratory	1.68 [0.96, 2.94]	1.83	0.0670
Kidney	1.72 [0.98, 3.04]	1.88	0.0603
Diabetes mellitus	2.04 [1.14, 3.68]	2.39	0.0169^*^
Hypertension	1.42 [0.69, 2.88]	0.96	0.3390
Gastrointestinal	1.24 [0.74, 2.07]	0.82	0.4101
**Medications**			
ACEI	0.95 [0.54, 1.68]	−0.18	0.8545
Calcium channel blockers	0.62 [0.36, 1.09]	−1.67	0.0947
Beta blockers	1.54 [1.12, 2.59]	1.65	0.0016^**^
Diuretics for heart failure	1.68 [0.77, 3.67]	1.31	0.1915
Diuretics for hypertension	0.92 [0.51, 1.67]	−0.27	0.7879
Antihypertensive drugs	0.77 [0.36, 1.68]	−0.66	0.5125
Antidiabetic drugs	0.39 [0.20, 1.79]	−2.61	0.242
**Complete blood count**			
Neutrophil, ×10^9^/L	0.98 [0.85, 1.14]	−0.21	0.8336
White cell count, ×10^9^/L	1.00 [0.87, 1.14]	−0.03	0.9728
Mean corpuscular hemoglobin, pg	1.07 [0.95, 1.21]	1.09	0.2749
Red blood count, ×10^12^/L	1.08 [0.58, 1.99]	0.23	0.8179
**Renal and liver function tests**			
Urate, mmol/L	0.21 [0.02, 2.51]	−1.24	0.2161
Albumin, g/L	1.00 [0.93, 1.07]	−0.14	0.8891
Urea, mmol/L	0.97 [0.83, 1.13]	−0.42	0.6770
Creatinine, μmol/L	1.00 [0.98, 1.01]	−0.65	0.5184
Alkaline phosphatase, U/L	0.99 [0.98, 1.00]	−1.44	0.1498
Bilirubin, μmol/L	1.02 [1.00, 1.04]	2.22	0.0264^*^
**Glycemic and lipid profiles**			
HDL, mmol/mol	1.00 [0.50, 1.98]	−0.01	0.9921
Fasting glucose, mmol/L	1.04 [0.98, 1.11]	1.32	0.1885
**Diastolic blood pressure measurements**			
Baseline, mmHg	1.03 [1.01, 1.07]	1.69	<0.0001^***^
Maximum, mmHg	0.98 [0.91, 1.06]	−0.48	0.6316
Minimal, mmHg	1.04 [1.01, 1.14]	0.90	0.0071^**^
Mean, mmHg	1.10 [1.02, 1.46]	0.61	0.0011^**^
Median, mmHg	0.91 [0.74, 1.12]	−0.9	0.3679
SD	0.70 [0.40, 1.24]	−1.23	0.2204
CV	1.05 [1.01, 1.12]	1.05	0.0659^*^
Variability score	1.02 [1.00, 1.04]	2.01	0.0047^**^
**Systolic blood pressure measurements**			
Baseline, mmHg	1.15 [1.08, 1.33]	0.40	<0.0001^***^
Latest, mmHg	1.01 [0.99, 1.03]	1.18	0.2389
Maximum, mmHg	1.00 [0.96, 1.04]	0.09	0.9302
Minimal, mmHg	1.04 [1.01, 1.19]	2.27	0.003^**^
Mean, mmHg	0.49 [0.02, 11.33]	−0.44	0.6579
Median, mmHg	1.04 [0.93, 1.16]	0.68	0.4949
Variance	1.00 [0.99, 1.01]	0.38	0.7017
SD	1.08 [0.70, 1.67]	0.36	0.7162
RMS	1.96 [0.09, 44.35]	0.42	0.6719
CV	1.04 [1.01, 1.09]	1.14	0.2540
Variability score	1.03 [1.01, 1.05]	1.27	0.7872

*ACEI, angiotensinogen-converting enzyme inhibitor; HDL, high-density lipoprotein cholesterol; SD, standard deviation; RMS, root mean square; CV, coefficient of variation*.

* *p ≤ 0.05*,

** *p ≤ 0.01*,

*** *p ≤ 0.001*.

### Comparisons of BP Measurements Before and After Anxiety Development

Gender-specific BP measure changes before and after initial anxiety presentation were identified as shown in Table 4. Regarding systolic BP, males had larger variance (median: 172.4, IQR: 99.5–262.7 vs. median: 165.7, IQR: 96.9–255.0, *p* < 0.0001), larger RMS (median: 137.1, IQR: 125.5–144.0 vs. median: 134.7, IQR: 126.2–143.0, *p* = 0.0489), and larger variability score (median: 73.6, IQR: 60.5–80.3 vs. median: 70.0, IQR: 60.0–77.0, *p* = 0.0332) after initial presentation of anxiety diseases; and females had larger variance (median: 174.2, IQR: 88.8–259.2 vs. median 161.8, IQR: 91.7–256.7, *p* < 0.0001) and larger variability score (median: 74.4, IQR: 60.5–79.3 vs. median: 70.6, IQR: 57.1–78.6, *p* = 0.0136) after initial presentation of anxiety diseases. In diastolic BP measurements, males had larger variance (median: 65.5, IQR: 30.3–81.0 vs. median: 54.3, IQR: 31.4–88.9, *p* < 0.0001) and larger variability score (median: 63.3, IQR: 40.2–71.2 vs. median: 57.1, IQR: 46.2–66.7, *p* < 0.0001) after initial presentation of anxiety; females had larger minimal test (median: 63.0, IQR: 54.0–67.0 vs. median: 58.0, IQR: 52.0–66.0, *p* = 0.0003), larger variance (median: 61.8, IQR: 31.4–81.4 vs. median: 56.1, IQR: 33.4–84.3, *p* < 0.0001), and larger variability score (median: 62.3, IQR: 43.2–67.54 vs. median: 55.8, IQR: 47.4–66.2, *p* < 0.0001).

**Table 4:** Gender-specific blood pressure measurement changes before and after anxiety development.

	**Males**			**Females**		
	**Before anxiety**	**After anxiety**	***P***	**Before anxiety**	**After anxiety**	***p***
**Systolic blood pressure measurements**						
Closest	133.0 (121.5–144.5); 211.0	136.0 (120.0–150.0); 217.0	0.1024	132.0 (119.0–144.0); 222.0	134.0 (120.0–148.0); 225.0	0.2226
Maximum	156.0 (144.0–169.0); 233.0	156.0 (143.0–170.0); 233.0	0.8723	157.0 (140.0–173.0); 246.0	156.0 (140.0–171.0); 246.0	0.2414
Minimal	111.0 (102.0–121.0); 164.0	112.0 (103.0–122.5); 173.0	0.7634	109.0 (101.0–120.0); 183.0	110.0 (102.0–121.0); 196.0	0.6821
Mean	134.0 (125.4–142.4); 191.6	135.5 (124.9–143.45); 187.0	0.2356	132.5 (123.3–141.0); 193.3	135.1 (122.9–143.0); 214.7	0.1386
Median	133.0 (125.0–142.0); 187.0	134.0 (123.25–143.0); 184.0	0.7631	132.0 (122.5–141.0); 195.0	135.8 (121.0–143.0); 223.0	0.0986
Variance	165.7 (96.9–255.0); 1,512.5	172.4 (99.5–262.7); 930.5	<0.0001^***^	161.8 (91.7–256.7); 2,964.5	174.2 (88.8–259.2); 2,056.2	<0.0001^***^
SD	12.9 (9.8–15.9); 38.9	13.1 (10.0–16.2); 30.5	0.4429	12.7 (9.6–16.0); 54.4	12.4 (9.4–16.1); 45.4	0.6199
RMS	134.7 (126.2–143.0); 192.4	137.1 (125.5–144.0); 187.4	0.0489^*^	133.4 (124.0–141.9); 193.6	135.7 (123.6–143.7); 215.1	0.1332
CV	0.09 (0.07–0.11); 0.2	0.09 (0.07–0.1); 0.2	0.9125	0.09 (0.07–0.12); 0.27	0.09 (0.07–0.11); 0.28	0.2305
Variability score	70.0 (60.0–77.0); 94.4	73.6 (60.5–80.3); 90.4	0.0332^*^	70.6 (57.1–78.6); 93.8	74.4 (60.5–79.3); 90.5	0.0136^*^
**Diastolic blood pressure measurements**						
Closest	75.0 (67.0–82.0); 135.0	77.0 (68.0–84.0); 122.0	0.1853	71.0 (63.0–79.0); 115.0	72.0 (65.0–80.0); 110.0	0.0461
Maximum	89.0 (81.5–97.0); 135.0	88.0 (80.0–96.0); 135.0	0.2296	86.0 (79.0–93.0); 128.0	85.0 (78.0–91.0); 122.0	0.7141
Minimal	63.0 (56.0–70.0); 100.0	65.0 (57.0–70.0); 100.0	0.1777	58.0 (52.0–66.0); 98.0	63.0 (54.0–67.0); 90.0	0.0003^***^
Mean	75.4 (69.8–81.6); 106.4286	75.5 (69.5–81.3); 106.3	0.5467	71.9 (66.2–77.2); 102.0	72.3 (66.3–77.4); 98.8	0.7072
Median	75.5 (69.5–81.0); 110.0	75.0 (69.0–81.0); 109.0	0.6943	71.5 (66.0–77.0); 102.0	72.0 (66.0–77.8); 97.5	0.0968
Variance	54.3 (31.4–88.9); 512.0	65.5 (30.3–81.0); 273.3	<0.0001^***^	56.1 (33.4–84.3); 924.5	61.8 (31.4–81.4); 690.3	<0.0001^***^
SD	7.4 (5.6–9.4); 22.6	7.0 (5.5–9.0); 16.5	0.4272	7.5 (5.8–9.2); 30.4	7.7 (5.6–9.0); 26.3	0.3177
RMS	75.9 (70.3–81.9); 106.6	75.7 (69.9–81.7); 106.43	0.7934	72.3 (66.6–77.6); 102.1	73.7 (66.8–77.7); 99.6	0.7846
CV	0.09 (0.07–0.12); 0.25	0.09 (0.07–0.13); 0.2	0.3691	0.1001 (0.073–0.1); 0.31	0.095 (0.07–0.12); 0.3	0.1045
Variability score	57.1 (46.2–66.7); 93.8	63.3 (40.2–71.2); 93.2	<0.0001^***^	55.8 (47.4–66.2); 92.3	62.3 (43.2–67.54); 90.5	<0.0001^***^

*SD, standard deviation; RMS, root mean square; CV, coefficient of variation*.

* *p ≤ 0.05*,

** *p ≤ 0.01*,

*** *p ≤ 0.001*.

## Discussion

The main findings of this study are that (1) higher baseline, maximum, minimum, SD, CV, and variability score of diastolic BP significantly predicted anxiety, as did all systolic BP measures (baseline, latest, maximum, minimum, mean, median, variance, SD, RMS, CV, and variability score) and (2) female and older patients with higher BP and higher BPV were at the greatest risks of anxiety.

The effects of anxiety on BP and as a potential risk factor have been extensively examined in previous studies (14). However, whether BP influences the risk of incident anxiety has not been investigated in detail, and mixed results were seen in observational studies. Individuals with hypertension may be more likely to develop anxiety (14, 15), but this association is seen only when hypertension coexists with another chronic condition (16) or when the patients are aware of their hypertension diagnosis (17). Previously, higher beat-to-beat BPV has been associated with incident anxiety (3). Longer-term visit-to-visit BPV has also been reported as an independent predictor of neurological conditions such as cognitive impairment in cohort studies (4–6), but whether it can do so for incident anxiety has never been explored. In this population-based study of patients attending family medicine clinics in the public sector of Hong Kong, we established for the first time the predictive value of different metrics of BP and BPV on incident anxiety.

While the physiological mechanisms underlying the bidirectional relationship between hypertension and incident anxiety remain unclear, the phenomenon was reported in recent studies. Population-based studies demonstrated that patients with baseline anxiety had an increased risk of essential hypertension (18–20). By contrast, a territory-wide study of over two million patients in Sweden demonstrated that hypertensive patients were more likely to suffer from anxiety (21). The presence of anxiety increases the risk of poor drug compliance among hypertensive patients and thus worsens their BP control (22).

Various hypotheses have been proposed to explain the association between anxiety and hypertension. First of all, chronic stress, which induces a persistent maladaptive stress response that develops into anxiety, results in long-term cortisol elevation (23). Consequently, the hypothalamic–pituitary–adrenal axis becomes dysregulated and leads to hypertension (24). Furthermore, it is postulated that exaggerated neurobiological sensitivity toward threat results in prolonged activation of the hypothalamic–pituitary–adrenal axis, which results in both the autonomic dysregulation underlying hypertension and the biological change under anxiety (25). Other mechanisms including increased oxidative stress, physical inactivity, and hypercapnia were reported to be common in both the pathogenesis of hypertension and anxiety and thus may contribute to the association between the two diseases (26–28).

Similarly, hypotheses have been proposed to explain the predictive value of BPV for incident anxiety. Increased BPV has been shown to be due to reduced baroreflex sensitivity, which may reflect sympathovagal imbalance likely due to sympathetic hyperactivity, which is observed in anxiety patients (3, 29, 30). The BP instability may reflect compensatory hemodynamic changes under reduced arterial compliance and increased aortic stiffness under systemic inflammatory response, which is both a cause of hypertension and a consequence of anxiety (31, 32). Furthermore, the pathological worrying in anxiety may be associated with increased compliance toward antihypertensives, which are known to increase BPV (33). Moreover, the use of medications such as beta blockers also predicted incident anxiety. It may be that anxious patients are more likely to receive such medications to reduce the symptoms of anxiety (34).

### Limitations

Several limitations should be noted for the present study. Given its observational nature, there is information bias with regard to issues of under-coding, missing data, and documentation errors. Moreover, data on lifestyle risk factors of hypertension, such as smoking and alcoholism, were unavailable; thus, their potential influence on the relationship between BP and anxiety cannot be assessed. Furthermore, the clinical circumstances of BP measurement during hospital visits were susceptible to the effects of circumstantial factors, which may introduce additional variables that affect patients' BP and BPV. Circadian changes in BP may be a good predictor of the adverse outcomes. However, our BP values were measured within the clinical setting. It was therefore not possible to obtain BP values at nighttime. Heart rate variability is also an important predictor, and this should be evaluated for its predictive value and incorporated into predictive risk models in subsequent studies. Finally, the diagnosis of anxiety was reliant on ICD-9 coding, and therefore, not all diagnoses were made by a specialist in psychiatry. However, results of psychological tools such as Generalized Anxiety Disorder 7 and Beck Anxiety Inventory are not routinely coded, and it was therefore not possible to precisely define the presence of anxiety disorder that fulfills the specialist definition of this disease.

## Conclusions

The relationships between longer-term visit-to-visit BPV and incident anxiety were identified. Female and older patients with higher BP and higher BPV were at the highest risks of anxiety. Future studies should examine the interacting effects between BPV and medication use to influence incident anxiety and anxiety-related outcomes.

## Data Availability Statement

The raw data supporting the conclusions of this article will be made available by the authors, without undue reservation.

## Ethics Statement

The studies involving human participants were reviewed and approved by The Joint Chinese University of Hong Kong—New Territories East Cluster Clinical Research Ethics Committee and Institutional Review Board of the University of Hong Kong/Hospital Authority Hong Kong West Cluster. Written informed consent for participation was not required for this study in accordance with the national legislation and the institutional requirements.

## Author Contributions

JZ and SL: data analysis, data interpretation, statistical analysis, manuscript drafting, and critical revision of the manuscript. WW, KL, RN, PL, and TL: project planning, data acquisition, data interpretation, and critical revision of manuscript. BC: study supervision, data interpretation, statistical analysis, and critical revision of manuscript. QZ and GT: study conception, study supervision, project planning, data interpretation, statistical analysis, manuscript drafting, and critical revision of the manuscript. All authors contributed to the article and approved the submitted version.

## Conflict of Interest

The authors declare that the research was conducted in the absence of any commercial or financial relationships that could be construed as a potential conflict of interest.
